# Mega-Analysis of Gene Expression in Mouse Models of Alzheimer’s Disease

**DOI:** 10.1523/ENEURO.0226-19.2019

**Published:** 2019-12-02

**Authors:** Beryl Zhuang, B. Ogan Mancarci, Lilah Toker, Paul Pavlidis

**Affiliations:** 1 Graduate Program in Bioinformatics, University of British Columbia, Vancouver V6T1Z4, Canada; 2 Department of Psychiatry, University of British Columbia, Vancouver V6T1Z4, Canada; 3 Michael Smith Laboratories, University of British Columbia, Vancouver V6T1Z4, Canada

**Keywords:** Alzheimer’s, bioinformatics, genomics, metaanalysis, transcriptome

## Abstract

While multiple studies have been conducted of gene expression in mouse models of Alzheimer’s disease (AD), their findings have not reached a clear consensus and have not accounted for the potentially confounding effects of changes in cellular composition. To help address this gap, we conducted a re-analysis based meta-analysis (mega-analysis) of ten independent studies of hippocampal gene expression in mouse models of AD. We used estimates of cellular composition as covariates in statistical models aimed to identify genes differentially expressed (DE) at either early or late stages of progression. Our analysis revealed changes in gene expression at early phases shared across studies, including dysregulation of genes involved in cholesterol biosynthesis and the complement system. Expression changes at later stages were dominated by cellular compositional effects. Thus, despite the considerable heterogeneity of the mouse models, we identified common patterns that may contribute to our understanding of AD etiology. Our work also highlights the importance of controlling for cellular composition effects in genomics studies of neurodegeneration.

## Abstract (precis)

While multiple studies have been conducted of gene expression in mouse models of Alzheimer’s disease (AD), their findings have not reached a clear consensus and have not accounted for the potentially confounding effects of changes in cellular composition. To help address this gap, we conducted a re-analysis based meta-analysis (mega-analysis) of ten independent studies of hippocampal gene expression in mouse models of AD.

## Significance Statement

A molecular understanding of Alzheimer’s disease (AD) is important to the development of treatments. Because of the difficulty of studying brain tissue in humans, especially at very early stages of progression, many groups have performed transcriptomic studies of rodent models. Our study identified changes in gene expression that are strikingly consistent across multiple such studies, providing substantial insight into molecular changes present at early stages of these disease models and which may be of importance in the human condition. Our study also demonstrates the importance of accounting for the complex effects of neurodegeneration on brain tissue in data analysis.

## Introduction

There is intense interest in understanding the molecular mechanisms that contribute to Alzheimer’s disease (AD), which involve complex interplays of genetic and environmental factors. However, the early changes in the brain before the onset of cognitive impairment in AD are still poorly understood (Masters et al., 2015). In AD patients, pathologic changes in the brain often precede the occurrence of clinical symptoms by years (Pennanen et al., 2004; Webster et al., 2014; Wirz et al., 2014). Postmortem brain samples of AD patients represent a late phase of the disorder, and it is challenging to distinguish causes from effects of neurodegeneration. Studying early molecular changes in mouse models improves our understanding of human disease pathogenesis. Various transgenic mouse models have been developed based on known mutations in familial AD cases and other AD related genes (Webster et al., 2014). Despite the differences in mechanisms, many of these animal models show similar age-dependent progression in decline of cognitive functions that are similar to AD symptoms observed in human (Webster et al., 2014). Identifying genes that are differentially expressed (DE) during early disease phase in the mouse models versus wild type could help understanding early responses to disease initiation, which may be neuroprotective or contribute to disease progression.

Recent expression profiling studies of AD mouse models have reported up-regulation of genes in classical complement cascade and changes of synapse-related genes in early AD disease progression (D’Onofrio et al., 2011; Hong et al., 2013, 2016). Studies of AD mouse models in later phases of progression (Matarin et al., 2015; Saura et al., 2015) often report expression changes of genes related to inflammation and the immune system. However, their findings are not completely concordant. Differences in mouse models can be one of the contributing factors, but differences in experiment design, sample size, microarray platforms, and data processing methods can also influence the results. Since these mouse models develop similar phenotypes, they could share molecular commonalities. A combined analysis of gene expression profiles of mouse models (which we refer to as a mega-analysis, to distinguish it from a meta-analysis, which traditionally refers to pooling of reported findings from studies rather than re-analyzing the raw data) has the potential to identify cross-mouse-model and disease-phase-specific transcriptional changes.

One of the challenges of bulk tissue transcriptome profiling in AD studies is that samples represent a weighted average of cellular composition, and cell counts of different cell types are typically not directly assessed. As a neurodegenerative disorder (ND), AD is characterized by neuronal loss and neuroinflammation (Serrano-Pozo et al., 2011). In AD patients and in some mouse models, loss of pyramidal neurons and other types of neurons in the hippocampus contributes to neuronal loss (Saxena and Caroni, 2011; Serrano-Pozo et al., 2011). Cell-type proportion changes have been identified as one of the driving forces for expression changes in bulk tissues of an AD mouse model (Srinivasan et al., 2016). Therefore, both cellular composition differences and cell type-specific expression differences can contribute to the overall expression changes in bulk tissue samples between disease models and controls, especially at late stages of progression. In contrast to late stages, early effects are expected to be relatively subtle but perhaps more informative about processes leading to pathology.

A recent study examined expression changes of neuronal-specific marker genes in whole brain tissues of human and mouse models of AD and revealed that expression profiles reflect cell population changes (Hokama et al., 2014). To address the cellular composition problem, methods for estimating cell-type proportions from bulk tissue transcriptome profiles have been developed, sometimes referred to as cell-type deconvolution. A commonly used method to deconvolute effects of cell-type proportion changes of bulk tissue is to estimate cell-type proportions by cell type-specific marker genes (Gaujoux and Seoighe, 2013; Shen-Orr and Gaujoux, 2013; Chikina et al., 2015; Mancarci et al., 2017). While not without caveats, accounting for cell type-specific expression changes improves the interpretability of bulk tissue datasets (Mancarci et al., 2017; Toker et al., 2018).

In this study, we re-analyzed publicly available expression data from the hippocampus of multiple AD mouse models, accounting for between-study differences and cell type-specific effects, with the goal of identifying consistent cross-mouse-model transcriptional changes, especially at early phases.

## Materials and Methods

Analyses were implemented in R (Team, 2016) except where noted. The data and scripts used are available at https://github.com/PavlidisLab/AlzMouseModelMeta.

### Data pre-processing and quality control

We retrieved gene expression profiling studies of mouse models of AD with the keyword “Alzheimer” from Gene Expression Omnibus (GEO; RRID:SCR_005012) and ArrayExpress (Barrett et al., 2013; Kolesnikov et al., 2015; RRID:SCR_002964). We further filtered the datasets and selected studies that have at least two biological replicate per condition and contained hippocampal samples. Initially 11 independent studies met the selection criteria. All were conducted on microarray platforms. We downloaded expression data and experimental design meta data from GEO. Probeset and gene annotations of the corresponding Affymetrix, Illumina and Agilent platforms were obtained from Gemma (Zoubarev et al., 2012; RRID:SCR_008007). On further examination, dataset GSE36981 was removed because genotype was confounded with sample batches. This left a final group of 10 datasets. Four major types of AD mouse models are among the 10: amyloid transgenic models, tau transgenic models, knock-out (KO) models and anti-NGF AD11 (categorized as “other”). Individual studies varied in their use of male or female animals, with both sexes included in the aggregated data. For overviews of the datasets, see Tables 1, 2.

**Table 1.** Summary of selected gene expression profiling studies for AD

Disease	Phase	Number of studies	Number of mouse models	Number of controls	Number of disease samples	Total samples	Total unique genes^c^
AD	Early	4	9	61	69	116	10,853
	Late	8	8	50	55	92	10,366
Total		10^a^	12^b^	84^c^	124	208	11,071^d^

a Two AD studies are categorized in both early and late disease phases.

b There are shared mouse models between early and late phases, and across studies.

c A total of 27 control samples were used as age-matched controls in both early and late phases in dataset GSE64398.

d Total unique genes are counts after removing genes with low expression values.

**Table 2.** Details of analyzed AD mouse model studies

Model types	Mouse model(s)	Study (dataset)	Phase(s)	Samples	Platform	Genes	M/F
Amyloid	Tg2576	GSE36237 (Kleiman et al., 2010)	Early	16 (8/8)	GPL1261	18118	0/16
		GSE1556 (Stein et al., 2004)	Late	4 (2/2)	GPL81	8237	0/4
		GSE15056 (Pereson et al., 2009)	Late	4 (2/2)	GPL7202	19459	0/4
	5xFAD	GSE52022 (Noh et al., 2014)	Late	4 (2/2)	GPL1261	18118	0/4
		GSE50521 (Paesler et al., 2015)	Late	12 (6/6)	GPL6096	16743	8/4
	J20	GSE14499 (Nagahara et al., 2009)	Late	6 (2/4)	GPL1261	18118	2/4
Amyloid TAU	TAS10, TPM, TASTPM TAU	GSE64398 (Cummings et al., 2015; Matarin et al., 2015)	Early, late	108(39/69)	GPL6885	17339	108/0
TAU	rTg4510	GSE53480 (Polito et al., 2014)	Late	8 (4/4)	GPL1261	18118	5/3
KO	Aplp2 KO, App KO, App/Aplp2 double-conditional KO (NdC-KO)	GSE48622 (Li et al., 2010)	Early	16 (4/12)	GPL1261	18118	16/0
Other	Anti-NGF AD11 (AD11)	GSE63617 (D’Onofrio et al., 2011)	Early, late	30 (15/15)	GPL7042, GPL7202	19459	0/30

Number of control and case samples are shown in parentheses (control/case). Genes refer to the number of unique genes mapped. M/F: number of male and female samples. Amyloid: amyloid transgenic models; TAU: TAU transgenic models.

The quality of the raw expression data of Affymetrix arrays was evaluated as described in (Rogic et al., 2016). All hippocampal samples using Affymetrix arrays passed the quality control procedures. Such raw data quality control procedures were not available for Agilent and Illumina arrays. To standardize data processing, Affymetrix and Agilent arrays were robust multi-array average (RMA) background corrected by *affy* (Gautier et al., 2004) and *limma* (Ritchie et al., 2015) R packages (RRID:SCR_012835, RRID:SCR_010943 respectively) followed by quantile normalization and log_2_ transformation. Illumina arrays were quantile normalized and log_2_ transformed. Samples with brain tissues other than hippocampus were discarded after normalization. Samples that were outliers (two standard deviations away from the mean sample-to-sample Pearson correlation within a dataset) were removed and the remaining samples were batch-corrected for each dataset by *ComBat* (Johnson et al., 2007; RRID:SCR_010974) if batch information was available.

The time points when mouse models first develop phenotypes that are similar to the earliest clinical symptoms for diagnosis in AD were used to define early and late phases of AD. The mouse phenotypes of AD mouse models analyzed were based on the behavior data from original publications, or the publications cited in the original paper. Mild cognitive decline is an early diagnostic symptom in AD patients (Webster et al., 2014). Cognitive impairment is often assessed by water maze test in AD mouse models. Therefore, AD mouse samples that did not display impairment in memory and learning measured by water maze tests were categorized as early phase AD samples, while the rest as late phase AD samples. The final dataset constituted data for early time points with controls (116 samples) and late time points with controls (96 samples).

To allow cross-platform comparison, within each dataset, we removed non-specific probes (i.e., probes that mapped to multiple genes), probes that did not map to any genes, and probes that contained missing expression values in one or more samples. When more than one probe mapped to a gene, we retained only the probe with the highest median expression value to represent the mapped gene. Not all the genes are available on all platforms used by the studies; we selected genes that were present in more than at least 2/3 of the platforms as a compromise between maximizing the number of genes in the analysis and the requirement to have multiple measurements to perform a mega-analysis. For each disorder, two integrated datasets were created by combining samples across studies from each disease phase. Within each integrated dataset, gene expression values were quantile normalized to harmonize scales across studies. We then filtered each dataset to remove non-expressed genes. To set the threshold for filtering, we were guided by the expression level of sex-specific genes (Toker et al., 2016). The signal for sex-specific genes in the non-expressing sex (e.g., Y-linked genes in females) can be taken as a rough indicator of background levels. The median expression value of non-expressed sex-specific genes from all samples was 5.2, and thus, we filtered genes with expression value lower than 6 as a more stringent threshold. For the number of genes in each disease phase after gene filtering, see Table 1.

Most of AD mouse models analyzed in this project were transgenic mouse models with transgenes under the control of murine Thy1 tissue-specific regulatory elements. The microarray probesets mapped to these transgenes and the endogenous copy, which artificially increased the measured expression of *Thy1*. Therefore, *Thy1* was removed from the mega-analysis in AD mouse models.

### Estimation of cell-type proportion changes

Cell-type proportions of three glial cell types (microglia, astrocytes, oligodendrocytes) and three neuronal cell types (pyramidal cells, dentate granule cells, GABAergic cells) were estimated by marker gene profiles (MGPs) using pre-selected markers specific to the murine hippocampus (Mancarci et al., 2017). Expression of the marker genes were first corrected for between-study variation for each disease phase and then used as input for MGP estimation. For presentation MGPs were normalized to a range between 0 and 1, where the sample with the highest profile was assigned to 1 and the lowest was assigned to 0. Wilcoxon rank-sum test and computation of false discovery rates (FDRs) using the Benjamini–Hochberg procedure (Benjamini and Hochberg, 1995) were applied to test whether the profiles were significantly different between disease mouse models and controls for each cell type.

### Fitting linear mixed-effects models (LMMs) and jackknife procedure to rank genes

LMMs allow modeling multiple sources of variation, such as mouse model-specific effects, laboratory effects, and difference between disease models and controls (Demirkale et al., 2010). For each disease phase group, two LMMs were fitted for each gene using the “lmer” function in R package *lme4* version 1.1-12 (RRID:SCR_015654), via maximum likelihood estimation [lmer(REML = F); Bates et al., 2015]. The first LMM corrected for between-study variations without correction for the MGPs; the second LMM corrected for both between-study variations and MGPs. The *p* value for the significance of the fixed effect of disease state (i.e., disease and normal states) was obtained by the “anova” function in R package *stats* version 3.3.1 (Chambers and Hastie, 1991). Ranking of upregulated or downregulated genes was based on *p* values in ascending order and the direction of expression changes between mouse models and controls. FDR estimated by the Benjamini–Hochberg procedure was computed for each gene. Significantly DE genes had FDR < 0.05. A jackknife procedure was applied to yield more robust gene rankings.

### Functional enrichment analysis

Functional enrichment analysis was performed using the threshold-free precision-recall algorithm in ErmineJ version 3.0.2 (Gillis et al., 2010; RRID:SCR_006450) to determine enrichment of Gene Ontology (GO) terms for the ranked list of genes from the jackknife procedure. We used the multifunctionality-adjusted enrichment rankings provided by ErmineJ to reduce the distorting effect of highly annotated genes (Ballouz et al., 2016).

## Results

We reanalyzed a total of 208 gene expression profiles from 10 AD mouse model datasets (Tables 1, 2). With the goal of identifying shared transcriptional alterations among different mouse models in the early and late phases, two separate mega-analyses were conducted: analysis of samples from “early” time points (before most pathologic effects), and a later time point. An important feature of our analysis is the use of MGPs. Used directly, MGPs can be used as an estimate of relative cellular proportions (Mancarci et al., 2017). We also used MGPs as covariates in our linear models to help isolate changes due to regulation as opposed to changes in cellular makeup of the tissue.

### Estimation of cell-type proportion changes

**Figure 1.**
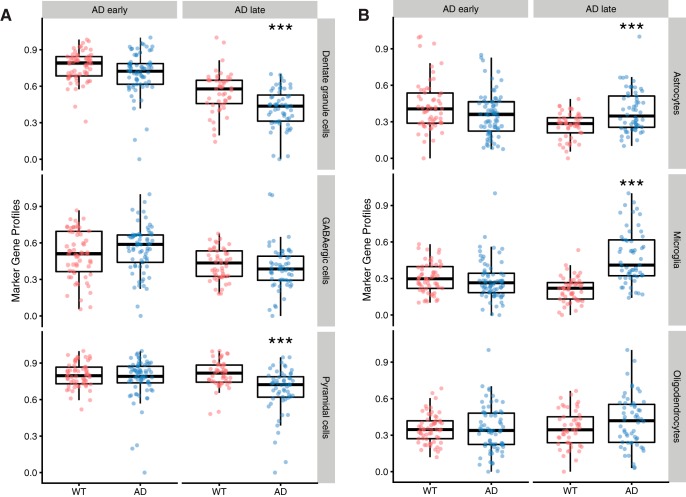
MGPs in AD mouse models. Each row of plots represents MGPs for one cell type as labeled at right. Each point represents one sample. Boxplots representing the interquartile range and the sample means are also shown. The vertical axis is normalized to the range 0 and 1. *** FDR < 0.01. ***A***, Neurons ***B***, Glial cells.

The results of MGP estimation were largely consistent with the expected cell-type changes in the early and late phase mouse samples. Relative to controls, mouse model samples in the early phase were predicted to show minimal or small cellular composition changes while late phase samples would show more substantial changes. In agreement, with this hypothesis, during the early phase, MGP analysis indicated no significant changes in neurons and glial cells (Fig. 1*A*,*B*). During the late phase, AD mouse models had estimated reduced dentate granule cells and pyramidal cells (Fig. 1*A*), and increased astrocytes and microglia (Fig. 1*B*).

### Mega-analysis of gene expression in AD mouse models

**Figure 2.**
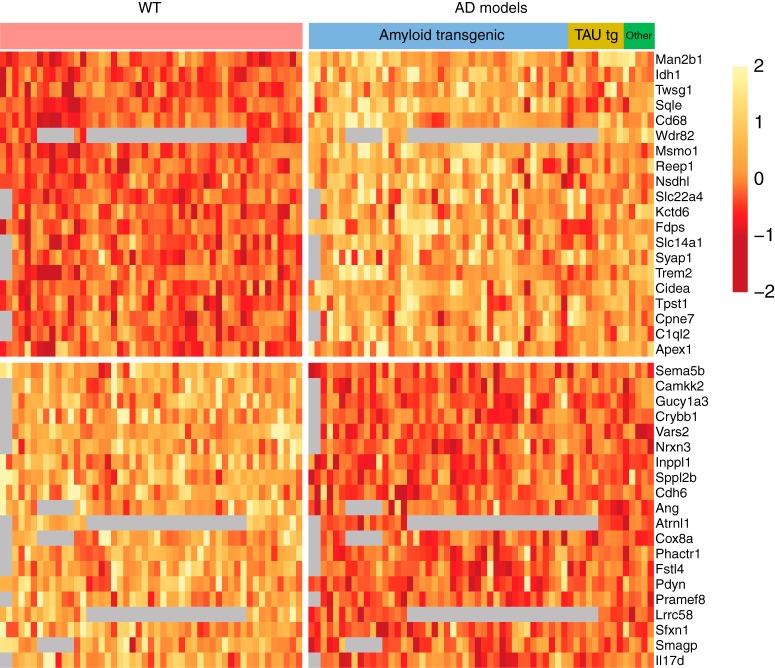
Top 20 upregulated and downregulated genes for late phase. Expression values are corrected for studies and MGPs. Each column is a brain sample, each row is a gene. For display purposes, each row is *z* score transformed. Gray cells represent missing values. A gap separates the upregulated genes (top) and the downregulated genes (bottom).

Using a mixed effect modeling approach with MGPs as covariates (see Materials and Methods), we identified expression changes associated with early and late phases of the AD mouse models. As expected, early changes were subtle. The mega-analysis of 116 gene expression profiles of mouse models and controls in the early phase revealed small but consistent expression changes (Fig. 2). Only four upregulated and three downregulated genes of the top genes were significant at an FDR of 0.05. A few of the top genes were functionally related based on the enrichment analysis. Three of the top 40 upregulated genes (*Sqle*, *Msmo1*, and *Nsdhl* ranked 2, 3, and 7; FDR < 0.05 except *Nsdh1*, which met an FDR of 0.08) are involved in cholesterol biosynthesis. *C1qa*, *C1qb*, and *C1qc*, which are involved in the classical complement cascade, were among the top 40 upregulated gene in the early phase (ranks 28, 12, and 29, meeting an FDR of 0.12).

**Figure 3.**
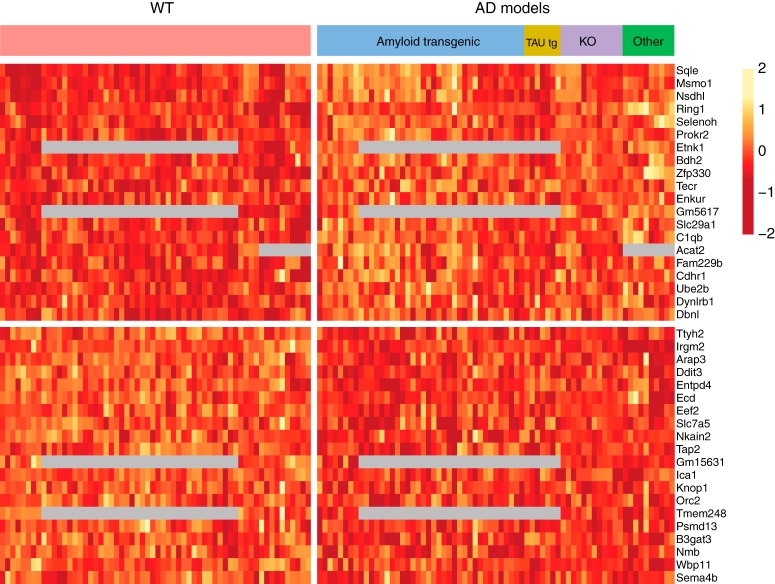
Top 20 upregulated and downregulated genes for early phase. As in Figure 2, but for the early phase.

In contrast to the subtle changes in the early phase, the expression change signals in the late phase were stronger (Fig. 3). The analysis also detected down-regulation of a known AD risk gene, *Trem2*, which had also been reported in the original publications of two studies analyzed. Several GO terms were found significantly enriched in the top downregulated genes, including “neuronal cell body” and “regulation of neurogenesis.”

We note that using MGPs as covariates have important effects on the results, which we documented by comparing the results of fitting models in which MGPs are not included. First, after MGP correction, as expected the marker genes included in the MGPs themselves are down-ranked, especially in the late phase. The number of significant DE genes (FDR < 0.05) in the late phase was greatly reduced after MGP correction:13.64% of the total genes were DE genes before correction and dropped to 0.22% after correction. We anticipated that the additional parameters included in the models would reduce power, so the fact that fewer genes met the FDR threshold was not in itself surprising. However, the gene rankings changed considerably, especially in the late phase. The Spearman correlation coefficients of before and after correction in the late phase is 0.38, compared to 0.80 in the early phase. Thus, MGP correction affected both the number of DE genes and the gene rankings, which indicated that cellular composition changes (as measured by MGPs) contributed to bulk tissue gene expression changes, especially in the late disease phase.

The gene rankings of early and late AD were quite different, indicating phase-specific effects. The Spearman correlation coefficient for gene ranking of all the genes between early and late phase was 0.24 before MGPs correction and 0.25 after. There were very few genes in common among the top 100 dysregulated genes across the phases. Specifically, only the top genes involved in cholesterol biosynthesis (*Sqle*, *Msmo1*, *Nsdhl*) were dysregulated in concordance in both phases (FDR < 0.05) for both before and after MGPs correction.

## Discussion

We identified consistent transcriptomic alterations across mouse models of AD. By categorizing samples into early and late disease phases, gene expression changes specific to the disease phase were revealed. This revealed subtle but biologically interpretable changes shared across mouse models in the early phase, which may reflect reveal early disease mechanisms. Changes in the late phase were stronger, and as expected were more associated with changes in cellular populations. The top-ranked genes in the early phase were not always affected in the late phase, and vice versa, indicating phase-specific expression changes.

### Consistent transcriptomic alterations were identified across different mouse models

**Figure 4.**
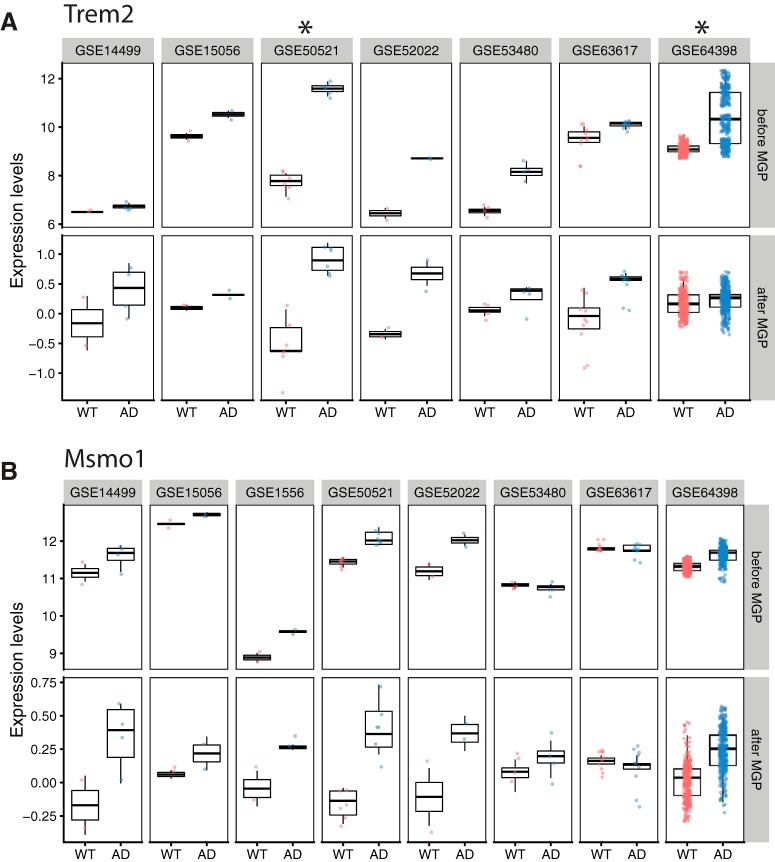
Examples of gene expression patterns identified. Each point represents one sample. Control samples (WT) are marked red and AD mouse model samples are blue. **“**After MGP**”** reflects analyses in which MGPs were included as covariates in the model; this correction rescales the expression levels to arbitrary units. Studies (datasets) that are marked with an asterisk have reported the gene as top hit in the original publications. ***A***, Expression of *Trem2* in the late AD phase before (top row) and after (bottom row) MGP correction. ***B***, Expressions of *Msmo1* in the late AD phase before (top) and after MGP correction (bottom).

Candidate genes identified in the original published reports were often inconsistent, though some genes are reported in more than one study. This may be due to differences in analysis methods and thresholds used, but doing a mega-analysis has the advantage of facilitating detection of weaker signals. Thus our mega-analysis was able to capture consistent signals that were not reported in the original source studies. For example, in the late AD phase, two out of seven studies reported the AD risk gene, *Trem2*, in their hit lists. However, we observed consistent up-regulation of *Trem2* in other studies (Fig. 4*A*). Similarly, we identified genes that had not been reported in any of the original studies, included a top upregulated gene, *Msmo1*, in the late AD phase (Fig. 4).

### Applying cell population proportion correction revealed transcriptional changes of cell type-specific regulatory events

We used MGPs to adjust the expression profiles for the effects of cellular proportion changes (Mancarci et al., 2017). We note that MGP analysis should interpreted as reflecting cellular proportions with caution, as it is an indirect measure. A more conservative interpretation of an MGP change is a cell type-specific change in expression. However, given the known neurodegeneration in these mouse models, changes in cellular proportions are the likely cause in MGP shifts in our study (Serrano-Pozo et al., 2011). Indeed, the cell-type proportion changes as estimated by MGPs in AD mouse models were consistent with previous mouse and human studies (Schmitz et al., 2004; Serrano-Pozo et al., 2011; Hokama et al., 2014), and indicated neuronal loss and gliosis in the hippocampus in the late phase (Fig. 1*A*,*B*). The lack of significant proportion changes of neurons and glial cells before the occurrence of cognitive impairment in AD mouse models (Fig. 1*A*,*B*) also agreed with a report that cognitive impairment, neuronal loss and gliosis occur concurrently in the hippocampus of AD mouse model J20 (Wright et al., 2013). Therefore, we interpret the MGP shifts as cellular proportion changes while keeping the indirect nature of the measure in mind.

The changes of gene rankings, and decreased DE signal after MGPs correction, implies that the gene expression changes, especially in the late disease phase, were substantially driven by changes in cell-type proportions. Adjusting MGPs can reveal cell type-specific transcriptional changes. Some of the markers remained top-ranked of the same direction of regulation even after correction, such as top-up-ranked microglia markers *Cd68* and *Tyrobp*, and astrocyte marker *Slc14a1* in the late AD phase remained top-up-ranked after correction. Because the dysregulation of these marker genes cannot be fully explained by the MGP changes, they may indicate changes at transcriptional regulation level within microglia that contribute to disease pathophysiology. However, because the changes in composition are confounded with the experimental condition, it is also possible that some residual effects of cellular proportion remain even after including MGPs as covariates. Ultimately resolution of cell type-specific changes in gene expression in these models, especially at late stages, will require cell type-specific transcriptomic studies.

### Shared gene expression changes revealed upregulation of genes in cholesterol biosynthesis and classical complement cascade in AD mouse models

Our mega-analysis of the early phase revealed up-regulation of genes in cholesterol biosynthesis and classical complement cascade. The changes were subtle in the early phase compared to the late phase. However, the results had some biological coherence as suggested by the GO enrichment analysis. Thus, the enrichment of genes involved in cholesterol biosynthesis in both early and late phases, even after MGP correction, suggested chronic dysregulation in cholesterol biosynthesis. Up-regulation of genes in cholesterol biosynthesis has been also observed in an AD amyloid transgenic mouse model, APP23 (Tseveleki et al., 2010). Many lines of evidence have linked cholesterol to AD, and to Aβ production in particular (Puglielli et al., 2003; Wood et al., 2014), though the interpretation and implications are still unclear. In mouse and cell culture studies, decreased brain cholesterol levels can reduce Aβ abundance (Di Paolo and Kim, 2011; Wood et al., 2014). Cleavage of APP by β-secretase and γ-secretase (the amyloidogenic pathway) mainly occurs in lipid rafts of the plasma membrane, whereas α-secretase of the non-amyloidogenic pathway tend to localize at the non-lipid-raft sites (Kim et al., 2016). Lipid rafts have high concentration of cholesterol (Kim et al., 2016). Increased level of cholesterol enhances localization of APP, β-secretase and γ-secretase to the lipid rafts, and subsequently promotes Aβ production (Marquer et al., 2011; Kim et al., 2016). However, the mechanisms that initiate the increased gene expression of cholesterol biosynthesis genes in mouse models are not known. The majority of samples analyzed here were amyloid transgenic models, which express human transgenes (*APP*, *PSEN1*, *PSEN2*) with known AD associated mutations that promote APP processing through the amyloidogenic pathway. Expression of these transgenes could play a role in promoting the expression of cholesterol biosynthesis genes, which lead to increased cholesterol level that can accelerate Aβ production. The most established AD risk factor is apolipoprotein E (ApoE, encoded by *APOE*), which mediates cholesterol metabolism in the brain and is found in Aβ plaques and neurofibrillary tangles (Yang and Song, 2013; Ries and Sastre, 2016). It has been shown that the high-risk isoform APOE4 has lower efficiency in transporting cholesterol from astrocytes to neurons compared to the neutral isoform APOE3, and lead to synaptic dysfunctions (Bu, 2009). Our results suggest cholesterol may play a role in AD-like process initiation and progression in mouse models.

Complement pathway genes, *C1qa*, *C1qb*, and *C1qc*, were among the top-ranked upregulated genes in the early phase, suggested up-regulation of genes in the C1q pathway in early disease progression. The up-regulation of C1q pathway, which initiates classical complement cascade, has been linked to early synaptic loss before Aβ accumulation, and is a response to injury in mouse model, and could be neuroprotective against misfolded proteins (Benoit et al., 2013; Hong et al., 2016). C1q pathway genes were still differentially upregulated in the late phase, however, they were no longer among the top-ranked genes after MGP correction. This suggests that cellular composition changes are more responsible for RNA levels changes of C1q genes in the late phase.

In the late phase, the GO term regulation of neurogenesis was enriched in the downregulated genes. The result was consistent with previous studies (Noh et al., 2014; Matarin et al., 2015), reflecting neuronal dysfunction in the AD mouse brain, though as noted above this could also potentially reflect imperfect correction for proportion effects. Interestingly, GO terms related to inflammation and the immune system were not enriched in upregulated genes. These results are contrary to other studies, which report up-regulations of genes related to inflammation and immune system (Matarin et al., 2015; Saura et al., 2015). This seeming discrepancy can be explained as a combination of effects of cell-type proportion considerations and gene function annotations. Without MGPs correction, our results did indicate up-regulation of genes in the immune response pathways. However, closer inspection revealed that some of the microglia markers are annotated with GO terms “immune response” and “inflammatory response.” This further complicates separating cell-type proportion changes regulatory effects. On the other hand, the dysregulation of a few astrocytes and microglia markers could not be fully explained by the cell-type population changes and remained top-ranked dysregulated genes after MGPs correction. For example, *Cd68*, a marker for microglial activation and is correlated with Aβ42 load (Zotova et al., 2011), was upregulated in the late phase. Another microglia marker, TYRO protein tyrosine kinase-binding protein gene (*Tyrobp*) and its receptor *Trem2* were among the top upregulated genes. A previous study reported that TYROBP binds to TREM2 and promotes microglial activation (Kobayashi et al., 2016). These results may provide some evidence of microglial activation at the cell type-specific level in AD mouse models.

In conclusion, our mega-analysis of gene expression in mouse models revealed consistent and disease phase-specific transcriptional changes and cell type-specific regulatory events, despite the considerable heterogeneity of the mouse models of AD. The identification of shared gene expression changes in the early phase increases our understanding of disease initiation and progression. Prioritized top-ranked genes in the early phase can be candidate genes to study mechanisms in disease initiation and worthy of further investigation.

## Synthesis

Reviewing Editor: Menno Witter, Norwegian University of Science and Technology Decisions are customarily a result of the Reviewing Editor and the peer reviewers coming together and discussing their recommendations until a consensus is reached. When revisions are invited, a fact-based synthesis statement explaining their decision and outlining what is needed to prepare a revision will be listed below. The following reviewer(s) agreed to reveal their identity: Peng Lei, Lene Olsen.

The manuscript entitled “Meta-analysis of gene expression in mouse models of Alzheimer's disease” has provided an interesting attempt to use bioinformatics for the understanding of pathogenesis of Alzheimer's disease. Such a meta-analysis of transcription data from the hippocampus of multiple animal models of Alzheimer's disease has not been done before. The authors report that consistent transcriptomic alterations across mouse early and late models of AD, identified genes that had not been reported yet and pointed the importance of MGPs.

The use of multiple types of animal models allows identification of common pathways involved resulting in similar pathology. The study differentiates sampling before and after onset of cognitive symptoms in the animals, which allows for identification of stage-specific molecular processes. The authors attempt to correct for the cellular composition effect, which is a well-known problem with bulk transcriptomics data on the disease due to loss of neurons and gliosis during disease progression. Indeed, they found that cell type composition dominates the transcription data at the later stages.

The main advance this study provides for the field is that a such systematic meta-study, if done correctly, might increase the power to detect differences between transgenic and wildtype models, and improve the validity of the findings of the studies included. In addition, the consistently differentially expressed genes they identified can be used as a resource for others in future studies on Alzheimer's disease.

We thus recognize the importance of meta-analysis on varies AD models but there are considerable doubts on how these models have been grouped and some other methodological issues raised by the reviewers.

1. The process of meta-analysis needs to be standardized. Although the authors gave the descriptions of data pre-processing and quality control, using flow charts to show the inclusion and exclusion process of original research for meta-analysis is necessary. They also need to state the methods for meta-analysis. Are they using combining P values and combining effect sizes? If properly described, the applied statistical data might be appropriate for the study and ensure robust findings across the data sets. However, due to journal policy, the code was not provided, so the reviewers could not determine that the analyses were conducted in an appropriate manner. Therefore, a short description in the methods section might be appropriate.

2. The current manuscript has presented the differences between early and late AD mouse model, which is interesting. However, it is important to provide a clear definition of early and late stage of AD.

3. The authors grouped varies models based on the presentation of pathology. However, what is the rational to combine amyloid pathology and tau pathology for any analysis? To perform such meta-analysis, the authors made the assumption that the pathologies were caused by the same process, which is not supported by the experimental evidences in the literature.

4. Many of ‘AD’ models used in this study is questionable. For example, AD11 anti-NGF mice is rarely considered as AD models. Introducing such model into the analysis could easily introduce more inflammatory factors that may not be of importance. Similarly, APP KO can hardly be considered as early stage of AD, nor does APLP2 KO.

5. Many of the studies included in the analysis only contain 2 animals. Is it statistically correct to include these data?

6. Is the categorization of late and early time points performed according to the phenotype of the particular animal model, or was a general time point set for all?

7. The paper only indicates QC for Affymetrix data. What about the data from other platforms?

8. What did the authors do with multiple probes covering the same gene?

9. It would be clearer for the reader if the authors specified at least once that they only used microarray and not sequencing data.

10. The authors state that only four genes were significantly upregulated at early time points after adjusting for multiple testing. However, they list three genes involved in cholesterol biosynthesis, and three in the complement cascade as being among the top upregulated results. Did they make some sort of cut off apart from the FDR < 0.05?

11. Some of the data sets contain testing of conditions other than just transgene vs. control mice. While the number of samples included in the table 2 indicates removal of such samples, it is not stated in the method section that such samples were removed.

12. A few mistakes in the text that warrant correction:

• There are some mistakes in the references, with the year being listed twice (e.g. on line 45)

• Mistake line 159: The word “Figur” should be deleted.

• In Table 1, the letter ‘c’ should be deleted from the header in the last column.

• Lines 196-199 states that Trem2 is downregulated, but that Msmo1 is upregulated. Figure 4 shows that these two genes are both upregulated in the Alzheimer's model mice compared to controls.
